# Comparative evaluation of behavior management techniques on anxiety levels in pediatric dental patients

**DOI:** 10.6026/973206300223240

**Published:** 2026-06-30

**Authors:** Jaya Verma, Ankita Verma, Jayesh Anand, Rishvee Prateek, Harsh Priyank, Asim Mustafa Khan, Haitham Ahmed Alhasan

**Affiliations:** 1 Department of Pedodontics and Preventive Dentistry, Project Research Scientist-2, ICMR- NIRBI, Kolkata, West Bengal, India; 2 Department of Pedodontics and Preventive Dentistry, Hazaribagh College of Dental Sciences and Hospital, Jharkhand, India; 3 Department or Oral and Maxillofacial Surgery, Hazaribag college of dental sciences and Hospital, Jharkhand, India; 4 Department of Prosthodontic and Implantogy, Hazaribag College of Dental Sciences and Hospital, Jharkhand, India; 5 Department of Conservative, Endodontics and Aesthetic Dentistry, Dental Institute, RIMS, Ranchi, India; 6 Department of Biomedical Dental Sciences, College of Dentistry, Imam Abdulrahman Bin Faisal University, Dammam, Saudi Arabia; 7 Department of Dentistry, College of Dentistry, Dar Al Uloom University, Riyadh, Saudi Arabia

**Keywords:** Pediatric dentistry, anxiety management, behavior management techniques (BMTs), nitrous oxide (NO), distraction (D), positive reinforcement (PR)

## Abstract

Dental anxiety in pediatric patients significantly hinders effective treatment, posing a barrier to optimal dental care. Therefore, it is of interest to evaluate the effectiveness of various behavior management techniques (BMTs) in reducing anxiety during dental procedures in children. Four BMTs were compared: Positive reinforcement, distraction, nitrous oxide sedation and control. Data showed that nitrous oxide sedation was the most effective, followed by distraction, while positive reinforcement offered moderate success. Thus, we show the most effective techniques for managing dental anxiety in pediatric patients.

## Background:

Dental anxiety in pediatric patients is a common and significant issue that poses a barrier to effective dental treatment and care. This anxiety often leads to avoidance of dental appointments, resulting in poorer oral health outcomes for children [1]. Behavioral management techniques (BMTs) play a crucial role in addressing dental anxiety in pediatric dentistry. These techniques aim to modify a child's behavior, enhance cooperation and reduce anxiety during dental procedures [2]. However, there remains a need for empirical evaluation of the effectiveness of various BMTs on reducing anxiety in pediatric dental patients [3]. Dental anxiety can be categorized into mild, moderate, or severe forms and the severity often correlates with the child's age, temperament, previous dental experiences and parental anxiety. It has been observed that anxiety levels can affect a child's ability to tolerate dental procedures, making it challenging for clinicians to deliver optimal care [4]. Furthermore, the emotional impact of early dental anxiety can extend into adulthood, potentially leading to long-term dental phobia. The etiology of dental anxiety is complex and may include factors such as fear of pain, unfamiliarity with the dental environment and past traumatic experiences during dental visits [5]. Behavior management strategies are designed to mitigate these anxieties by focusing on creating a more relaxed and cooperative environment. These strategies may range from non-pharmacological approaches, such as positive reinforcement, distraction and guided imagery, to more complex techniques like sedation or the use of nitrous oxide [6]. Positive reinforcement, which rewards desirable behavior, is one of the most widely used methods in pediatric dentistry. It helps to build trust and cooperation by reinforcing the child's positive response to dental procedures [7]. Another common technique is the use of distraction, where the child's attention is diverted from the procedure through visual or auditory stimuli. Studies have shown that such methods can be effective in reducing anxiety levels, especially in younger children who may find dental instruments and environments overwhelming [8]. Sedation techniques, including nitrous oxide, oral sedatives and conscious sedation, are often considered for children with moderate to severe anxiety. These approaches are more invasive than the non-pharmacological techniques but can be crucial for patients who exhibit significant distress or inability to cooperate during dental treatment [9]. Despite their success, these methods carry risks, including potential side effects and the need for careful monitoring during administration [10]. Given the widespread use of these techniques and the importance of effective management of dental anxiety in children, a more through and comparative evaluation is necessary to determine which methods offer the best outcomes for anxiety reduction [11]. Therefore, it is of interest to evaluate the effectiveness of different behavior management techniques and their impact on anxiety levels in pediatric dental patients.

## Methodology:

The study utilized a comparative design to evaluate the effectiveness of different behavior management techniques (BMTs) in reducing anxiety levels in pediatric dental patients. A total of 100 participants, aged between 4 and 12 years, were recruited from a pediatric dental clinic where they were scheduled for routine dental treatment. Ethical approval was obtained from the institutional review board (IRB) and informed consent was acquired from the parents or guardians of the participants. The inclusion criteria for the study required children to be aged between 4 and 12 years, either attending their first dental visit or undergoing routine procedures such as restorative treatments or scaling. Children with a history of significant medical conditions, prior traumatic dental experiences, or severe cognitive or physical disabilities were excluded from participation. In addition, children who had previously received any behavior management techniques or sedation for dental procedures were also excluded. Participants were randomly assigned to one of four groups, with each group receiving a different behavior management technique: Positive Reinforcement (PR), Distraction (D), Nitrous Oxide Sedation (NOS), or a Control Group (CG) receiving standard care. Each group consisted of 25 children. In the Positive Reinforcement group, children were rewarded with praise and small incentives for demonstrating appropriate behaviors during the dental procedure, such as remaining calm and following instructions. In the Distraction group, audiovisual aids such as cartoons or music, along with guided imagery techniques, were used to divert the child's attention away from the procedure. The Nitrous Oxide Sedation group received conscious sedation with nitrous oxide to help reduce anxiety and promote relaxation. The Control Group received only the standard dental care protocol without any specific behavioral management technique. Before the dental procedure, baseline anxiety levels were assessed using the Venham's Anxiety Scale, which was completed by the parents of the participants. Additionally, each child's heart rate and blood pressure were recorded to assess physiological indicators of anxiety. During the dental procedure, anxiety levels were measured using the Modified Child Dental Anxiety Scale (MCDAS), which was completed by the attending dentist and dental assistant based on the child's behavior. Signs of anxiety, such as restlessness, crying, or refusal to cooperate, were documented. The primary outcome measure was the reduction in anxiety levels observed during the dental procedure. These levels were assessed both through self-reports (if applicable) and observer ratings. Heart rate and blood pressure were monitored throughout the procedure, with any significant changes indicating an increase in anxiety. Following the procedure, the parents completed a follow-up questionnaire to evaluate their child's anxiety levels and behavior after the treatment. Data were analyzed using SPSS software. Descriptive statistics summarized the baseline characteristics of the participants. The primary analysis compared the anxiety levels between the four groups using ANOVA to identify any significant differences in anxiety reduction. Post-hoc analyses were performed if significant differences were found and a p-value of less than 0.05 was considered statistically significant. In conclusion, the study aimed to provide a comparative evaluation of various behavior management techniques to determine which approach was most effective in reducing anxiety in pediatric dental patients. The findings contributed to identifying the optimal strategies for managing anxiety and improving the dental care experience for children.

## Results:

The study evaluated and compared the effectiveness of four behavior management techniques-Positive Reinforcement, Distraction, Nitrous Oxide Sedation, and a Control approach-in reducing anxiety among pediatric dental patients undergoing dental procedures. Anxiety assessment was performed using both psychological scales and physiological parameters, enabling a comprehensive evaluation of behavioral and stress responses during treatment. At baseline, the anxiety levels of the participants were assessed using Venham's Anxiety Scale. As demonstrated in Table 1, the mean anxiety scores among the four groups were relatively similar, with scores ranging from 6.4 ± 1.1 in the Nitrous Oxide Sedation group to 6.7 ± 1.2 in the Control group. The Positive Reinforcement and Distraction groups showed mean baseline scores of 6.5 ± 1.2 and 6.6 ± 1.3, respectively. Statistical analysis revealed no significant differences between the groups prior to the intervention (p >0.05), indicating that all participants had comparable anxiety levels at the beginning of the study and that the groups were homogenous before treatment initiation (Table 1). Following the dental procedure, anxiety levels were reassessed using the Modified Child Dental Anxiety Scale (MCDAS). The findings revealed statistically significant differences among the groups after implementation of the respective behavior management techniques. As shown in Table 2, the Nitrous Oxide Sedation group demonstrated the lowest post-procedure anxiety score (2.5 ± 0.7), indicating the greatest reduction in anxiety. The Distraction group also exhibited a marked reduction in anxiety levels with a mean score of 3.8 ± 0.9, followed by the Positive Reinforcement group with a mean score of 4.2 ± 1.0. In contrast, the Control group showed the highest anxiety score post-treatment (5.6 ± 1.1), suggesting that routine dental management without a specific anxiety-reducing intervention was less effective in alleviating dental fear and apprehension among children (Table 2). Physiological indicators of stress and anxiety were further evaluated through changes in heart rate during the dental procedure. Table 3 illustrates that the Nitrous Oxide Sedation group exhibited the smallest mean increase in heart rate (4.5 ± 2.2 bpm), reflecting superior physiological relaxation and reduced autonomic stress response. The Distraction group demonstrated a moderate increase in heart rate (8.4 ± 2.9 bpm), while the Positive Reinforcement group showed a slightly greater increase (10.2 ± 3.5 bpm). The highest heart rate elevation was observed in the Control group (12.3 ± 4.1 bpm), indicating a greater anxiety response during dental treatment in the absence of a structured behavioral intervention (Table 3). Similarly, blood pressure changes recorded during the procedure supported the psychological findings. According to Table 4, the Nitrous Oxide Sedation group showed the least increase in systolic and diastolic blood pressure, with mean changes of 3.5 mmHg and 2.2 mmHg, respectively, accompanied by the lowest standard deviation (1.5). The Distraction group demonstrated comparatively lower blood pressure elevations (5.8 mmHg systolic and 3.1 mmHg diastolic) than the Positive Reinforcement group (6.3 mmHg systolic and 3.7 mmHg diastolic). Conversely, the Control group exhibited the highest increase in both systolic and diastolic blood pressure values (8.9 mmHg and 5.4 mmHg, respectively), indicating heightened physiological stress during the dental procedure (Table 4). Overall, the findings of the study demonstrated that Nitrous Oxide Sedation was the most effective behavior management technique for reducing both subjective anxiety levels and objective physiological stress indicators in pediatric dental patients. Distraction techniques also proved to be highly beneficial in minimizing dental anxiety, although their effectiveness was comparatively lower than nitrous oxide sedation. Positive Reinforcement showed moderate efficacy and contributed to anxiety reduction to a certain extent. In contrast, the Control group consistently exhibited the highest levels of anxiety and physiological stress responses across all evaluated parameters. These observations emphasize the importance of implementing appropriate behavior management strategies in pediatric dentistry to improve patient cooperation, reduce procedural stress, and enhance the overall dental experience for children. The results further suggest that the selection of a behavior management technique should be individualized according to the child's anxiety level, behavioral profile, and treatment requirements.

## Discussion:

The findings of the present study demonstrated that Nitrous Oxide Sedation was the most effective technique in reducing anxiety levels among pediatric dental patients, closely followed by Distraction and Positive Reinforcement, with the Control group showing the least reduction in anxiety. These results are consistent with previous research that highlights the efficacy of pharmacological sedation, particularly nitrous oxide, in pediatric dentistry. Xia *et al.*(2025) [12] reported that nitrous oxide significantly reduces anxiety and improves treatment success rates in children undergoing dental procedures, with minimal adverse effects, supporting our observation that sedation is highly effective in anxiety modulation. Moreover, Ghabchi *et al.*(2025) [13] found nitrous oxide-oxygen inhalation sedation to be a safe method for managing dental anxiety and facilitating cooperative behavior during dental care, further validating the effectiveness of nitrous oxide observed in our sample. In contrast, the enhanced performance of Distraction techniques in reducing anxiety aligns with the growing body of literature that emphasizes the role of non-pharmacological strategies. Chen *et al.*(2024) [14] highlighted that distraction methods using audiovisual and multisensory stimuli effectively divert children's attention away from anxiety-provoking stimuli, leading to lower anxiety and improved comfort during dental treatment. Similarly, Almaeen *et al.*(2025) [15] reported that distraction techniques were the most effective in reducing anxiety compared to tell-show-do and voice control, which parallels our finding that Distraction ranked superior among non-sedation strategies. These results suggest that while pharmacological sedation may yield the greatest reduction in objective anxiety measures, distraction remains a highly valuable, non-invasive alternative, particularly when sedation is not feasible or indicated. Positive Reinforcement also demonstrated significant benefits in improving behavior and lowering anxiety, which is consistent with findings in the pediatric dental literature. Verma *et al.*(2024) [16] showed that positive reinforcement resulted in high patient cooperation and treatment success rates compared with other behavior management methods, emphasizing its effectiveness in building trust and reinforcing desirable responses during dental treatment. Additionally, research summarized in a 2024 systematic review suggested that techniques rooted in behavioral psychology, such as live modeling and operant reinforcement, contribute significantly to anxiety reduction by engaging children's willingness to comply and perceive dental care positively [17]. Collectively, these studies confirm the value of positive reinforcement as a reliable behavior management tool, particularly for less invasive procedures or mildly anxious patients. However, while pharmacological approaches like nitrous oxide offer robust anxiety control, previous research has also noted potential limitations. For example, conscious sedation requires appropriate patient selection and careful monitoring and may not always be acceptable to all practitioners or parents. Samanta *et al.*(2025) [18] emphasized the rapid onset and effectiveness of nitrous oxide, but also underscored the need for proper training and precautions when using sedation in children. This highlights that while sedation is effective, optimal pediatric behavior management often involves a balanced integration of pharmacological and non-pharmacological techniques tailored to individual anxiety levels and clinical contexts.

## Conclusion:

We show that nitrous oxide sedation was the most effective technique in reducing anxiety levels in pediatric dental patients, followed by distraction and positive reinforcement. Data show the importance of tailoring behavior management strategies to individual patient needs. Therefore, both pharmacological and non-pharmacological techniques should be considered for optimal anxiety reduction in pediatric dental care.

## Figures and Tables

**Table 1:** Baseline anxiety levels of participants

**Group**	**Mean Anxiety Score**	**Standard Deviation**
Positive Reinforcement	6.5	1.2
Distraction	6.6	1.3
Nitrous Oxide Sedation	6.4	1.1
Control Group	6.7	1.2

**Table 2:** Post-Procedure anxiety levels measured by MCDAS

**Group**	**Mean Anxiety Score**	**Standard Deviation**
Positive Reinforcement	4.2	1
Distraction	3.8	0.9
Nitrous Oxide Sedation	2.5	0.7
Control Group	5.6	1.1

**Table 3:** Mean heart rate changes during the procedure

**Group**	**Mean Heart **	**Standard **
	Rate Change (bpm)	Deviation
Positive Reinforcement	10.2	3.5
Distraction	8.4	2.9
Nitrous Oxide Sedation	4.5	2.2
Control Group	12.3	4.1

**Table 4:** Mean blood pressure changes during the procedure

**Group**	**Systolic BP Change (mmHg)**	**Diastolic BP Change (mmHg)**	**Standard Deviation**
Positive Reinforcement	6.3	3.7	2.1
Distraction	5.8	3.1	1.9
Nitrous Oxide Sedation	3.5	2.2	1.5
Control Group	8.9	5.4	2.5
